# How the Number of Players and Floaters’ Positioning Changes the Offensive Performance during Futsal Small-Sided and Conditioned Games

**DOI:** 10.3390/ijerph18147557

**Published:** 2021-07-15

**Authors:** David Pizarro, Alba Práxedes, Bruno Travassos, Bruno Gonçalves, Alberto Moreno

**Affiliations:** 1 Faculty of Life Sciences and Nature, University of Nebrija, 28015 Madrid, Spain; apraxedes@nebrija.es; 2 Research Center in Sport Sciences, Health and Human Development (CIDESD), Department of Sport Sciences, University of Beira Interior, 6201-001 Covilhã, Portugal; BrunoTravassos@hotmail.com; 3 Portugal Football School, Portuguese Football Federation, 5001-801 Oeiras, Portugal; bgoncalves@uevora.pt; 4 Departamento de Desporto e Saúde, Escola de Saúde e Desenvolvimento Humano, Universidade de Évora, 7004-516 Évora, Portugal; 5 Comprehensive Health Research Centre (CHRC), Universidade de Évora, 7004-516 Évora, Portugal; 6 Faculty of Sport Sciences, University of Extremadura, 06006 Cáceres, Spain; amorenodfcd@gmail.com

**Keywords:** ecological dynamics, training tasks, technical–tactical training, game principles

## Abstract

This study aims to analyse the effects of floater positioning within futsal Gk + 3vs3 + Gk and Gk + 2vs2 + Gk small-sided and conditioned games (SSCG) on youth offensive performance on an action per minute per player basis. Three experimental conditions were carried out through the manipulation of floater positioning: floaters off (FO), final line floaters (FLF) and lateral floaters (LF). Thirty male futsal players (U19 age category) participated in the study and played once within each situation in a random order on different days. Offensive performance based on “action per minute per player” was analysed through indirect and external systematic observation. Results showed significant differences between both SSCGs (2vs2 and 3vs3). Specifically, according to the game principles analysed, 3vs3 is associated with higher values of passing and dribbling action to progress towards the goal without beating a defensive line (moderate to large effect size), while 2vs2 is associated with higher values of passing and dribbling actions that beating a defensive line (moderate to very large effect size). In addition, 2vs2 is associated with dribbling and shooting actions to shoot at goal with the lowest level of opposition (moderate effect size). Indeed, whilst the 2vs2 game format seems to promote more 1vs1 situations, the 3vs3 game format encourages more ball possession and collective tactical behaviours. Thus, training tasks intended to improve dribbling and shooting actions should use a smaller number of players whereas tasks intended to improve passing actions for ball possession should include a higher number of players with or without floaters. It seems that the number of players can influence the tactical behaviour of the team. These findings should be considered for the design of futsal training tasks, according to the main objective of the training session. For example, if the coach aims to promote the number of dribbles and shots within a SSCG, 2vs2 SSCG situations should be prioritised.

## 1. Introduction

In team sports such as futsal, in which open motor skills predominate, it is required that players continuously co-adapt their actions such as positioning, passes, dribbles, or shots to the movements of opponents, teammates, and the surrounding environment, leading to the emergence of opportunities for action [1,2,3] and to ensure functional collective behaviour [4,5,6]. In the last few decades, based on the ecological dynamics approach, non-linear pedagogy has emerged as a new teaching–learning perspective to promote a holistic approach that highlights the need to maintain the perception–action couple on the design of practice tasks [5]. For example, manipulating small-sided and conditioned games (SSCG), coaches can highlight the actions and the information that will support players’ performance. SSCG (commonly used modified games that take place in tight spaces, involving small numbers of players and with modified rules) are modified games that optimize the physical, physiological, technical, and tactical demands of sports instead of replicating a real match [7]. However, the advantages of playing SSCG are dependent on the task’s goals and design [8] that guides players to explore the functional behaviours of each task according to the coaches’ primary purposes [9].

The manipulation of task constraints in SSCG seems to be an effective approach to skill acquisition [10] that allows coaches to optimize specific offensive or defensive behaviours of players by breaking the game into specific game subunits, i.e., Gk + 1vs1 + Gk until Gk + 3vs3 + Gk [11]. This is likely to maintain the perceptual-motor demands of the match and the spatial-temporal relations between teammates and opponents, instead of replicating the technical and tactical demands of sports [7]. Indeed, coaches should go from simplified units with a low number of players to highlight the informational constraints that promote the development of offensive or defensive foundations of players to more complex units until the numerical relation of the game to develop the game principles and strategic requirements that support collective behaviour of teams according to the perceptual and action demands of competition [11].

Previous studies have attempted to provide a broader comprehension of the impact of altering SSCG characteristics (task constraints), such as the number of players per team [12,13], the court size [1], number of targets [14] or even the manipulation of the numerical relation between teams using floaters (jokers in other studies) [15,16,17,18,19,20,21]. Interestingly, one of the task constraints that have been studied recently is the accomplishing of tactical principles of attack performance [22]. These are referred to as keep possession, progress towards the goal (with or without beating a defensive line), or shoot at goal with the lowest level of opposition [23]. For example, regarding the manipulation of floaters, previous studies suggested that the use of on-field floaters increased players’ decision-making efficiency due to their distribution over the breadth of the field [24]. Moreover, on-field floaters might have afforded more opportunities for passing the ball, allowing the team to maintain ball possession [15]. Hence, manipulation of the relevant task constraints (e.g., presence of floaters and their positioning) for each goal can guide players to explore the environment of play, improving their tactical and creative behaviour [25].

The effects of such manipulations to design appropriate learning environments that help players’ development of adaptative technical–tactical behaviours to changes in the game environment [8] must be well understood by coaches, particularly in futsal. This perspective explains the interest of researchers and practitioners in this topic and the growing number of studies over the last few years [7,26,27]. However, no information exists regarding the technical–tactical changes promoted by the manipulation of the number of players and floater positioning. Thus, the main purpose of this study was to analyse the manipulation on the number of players (Gk + 3vs3 + Gk and Gk + 2vs2 + Gk) and floater positioning on youth players’ technical–tactical offensive actions according to game principles.

## 2. Materials and Methods

### 2.1. Participants

Thirty male futsal players from the under-19 (U19) category (age, M = 17.714 and SD = 0.713) of four different Spanish clubs agreed to participate in this study. All the participants had the same level of expertise (i.e., average skill level) and participated in the same competition (the first regional league). All teams had the same amount of training (i.e., players perform two training sessions of 60 min per week with an official match played on weekends).

### 2.2. Design and Procedures

**Figure 1:**
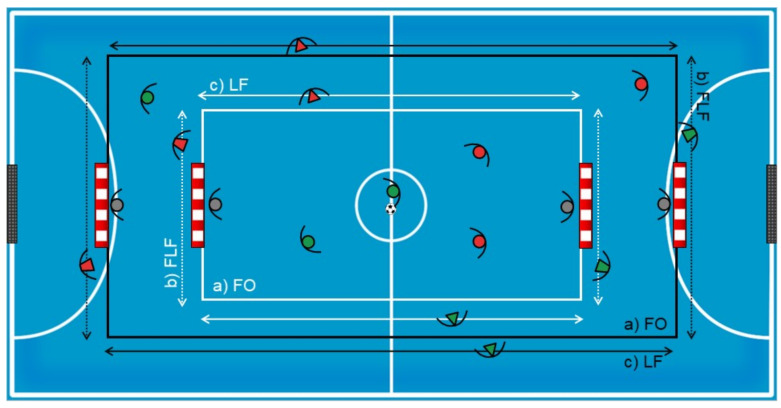
3vs3 and 2vs2 experimental conditions. (**a**) FO: Floaters Off; (**b**) FLF: Final Line Floaters; (**c**) LF: Lateral Floaters.

The study designed consisted of an independent measure approach under three experimental conditions (three SSCG) that manipulated the floater positioning. These SSCG (Gk + 3vs3 + Gk; Gk + 2vs2 + Gk) were designed using the presence and absence of “Floaters” (2 Floaters; one per team) as key task constraints: (a) “Floaters Off” (FO); (b) “Final Line Floaters” (FLF) (associated with the 3-1 offensive system) and (c) “Lateral Floaters” (LF) (associated with the 4-0 offensive system). In 3vs3 situations, tests were conducted on a 30 m long by 15 m wide field. In 2vs2 situations, tests were conducted on a 20 m long by 10 m wide field (see Figure 1). These measures respected the player-space ratio used by futsal players according to the maximum length and width dimensions (40 m × 20 m) of the real game (for each team player, 10 m large and 5 m regular, excluding goalkeepers).

Players were randomly distributed into five groups of six individuals for 3vs3 SSCG (G1 to G5); seven groups of four individuals for 2vs2 SSCG (G1 to G7, two players were randomly excluded for 2vs2; goalkeepers and floaters with goalkeepers and floaters no measurements were taken). All participants played once in each situation in random order and on a different day. Each 3vs3 testing had the following organization: warm-up (12′) + SSCG of 12′: 3′-1′-3′-1′-3′-1′ (3′ period = playing; 1′ period = resting); and 2vs2 testing: warm-up (10′) + SSCG of 10′: 2′-1′-2′-1′-2′-1′ (2′ period = playing; 1′ period = resting). During the rest intervals between bouts, players could drink water.

Game situations were explained, and participants were asked to play at their best level to succeed in the SSCG (score in the opposite goal). Coaches and experimenters did not provide any verbal feedback during the SSCG. Floater players were only allowed to perform offensive actions, with a maximum of two touches, and their actions were limited to the space between two marks parallel to each line (side or final) and could not score a goal. In addition, goalkeepers could not leave the goal line, and a throw-in was granted after the ball crossed the lines delimiting the floaters’ area. During the test, players were asked not to go inside the floaters’ area and balls were placed around the field to allow a quick restart of the game if the ball went out of play. In between bouts, players were allowed to drink water.

### 2.3. Data Collection

**Figure 2:**
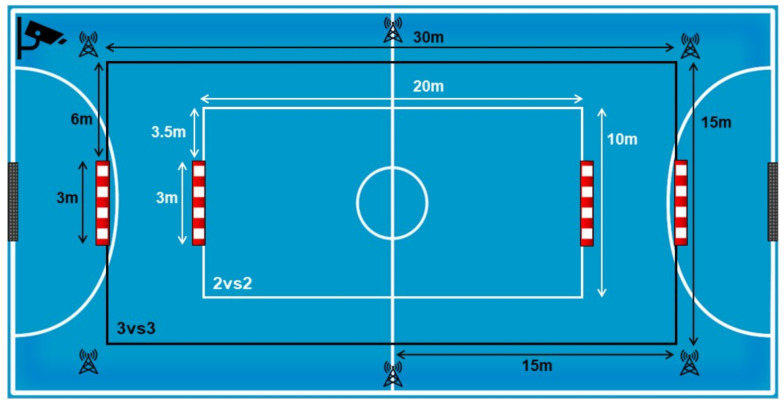
Pitch size and camera positioning.

All game actions within SSCGs were recorded using a video camera, recording angle conversion lens (×0.75): VCL-HGA07B and a Hama Gamma tripod Series. The camera was placed in the corner of the playing field, at the height of 4 m, guaranteeing an optimal view of all the game actions (see Figure 2). Videos were transferred to a computer (Acer Aspire E15). Subsequently, data were recorded on a Microsoft Office Excel 2010 sheet and exported to SPSS Inc., Released 2009 (PASW Statistics for Windows, Version 18.0, Page: 4 SPSS Inc., Chicago, IL, USA). Offensive performance measured as “action per minute per player” was analysed through indirect and external systematic observation, a methodology used in previous studies to measure players’ behaviour in real game situations [28].

Two external researchers conducted the observations. As a preparatory stage to the observations, the expert met with the observer to clarify possible doubts about the observation instrument and the coding criteria of the dependent variable on the actions mentioned. Then, the observations were carried out using more than 10% of the sample (*n* = xxx) [29]. Interobserver reliability was calculated using the following formula: agreements/(agreements + disagreements) × 100. Once this value was calculated, the Cohen kappa index was used. Values above 0.90 were obtained for all training sessions, surpassing the value of 0.81 from which adequate concordance is considered [30], thus achieving the necessary reliability for the subsequent coding of the dependent variables. To guarantee the time reliability of the measurement, the same coding was performed at two different moments, with a time difference of 10 days. Cohen kappa values were found to be higher than 0.92, which reflected a reliable concordance.

All the passing, dribbling, and shooting actions of each player in the team were analysed according to the following game principles: 1st principle—to keep possession (BP) (for passing and dribbling, when the action was developed horizontally or backwards); 2nd A principle—to progress towards the goal without beating a defensive line (P) (for passing and dribbling, when the action developed was forward, not beating a defensive line); 2nd B principle—to progress towards the goal beating a defensive line (PDL) (for passing and dribbling, when the action developed was forward, beating a defensive line); 3rd principle—to shoot at goal with the lowest level of opposition (S) (for passing and dribbling, when the action ended at the first touch after it, regardless of its direction; and for any shooting action).

### 2.4. Statistical Analysis

The statistical analysis was completed using The Jamovi Project (Jamovi). A descriptive analysis is presented on Table 1, with mean and standard deviation (Mean ± SD). An independent sample *t*-test was performed to identify differences in considered variables between the game formats 2vs2 vs. 3vs3. Statistical significance was set at *p* < 0.05. Additionally, to overcome the shortcomings associated with traditional N-P null hypothesis significance testing, the standardized Cohen’s d, with 95% confidence intervals were used as the effect size (ES) of the differences [31,32,33]. Thresholds for effect size statistics were: 0.0–0.19, trivial; 0.20–0.59, small; 0.6–1.19, moderate; 1.2–1.99, large; and ≥2.0, very large [33].

## 3. Results

For 3vs3 SSCG, a total of 1352 passing (1st principle, n = 573; 2nd A principle, *n* = 548, 2nd B principle, *n* = 127; 3rd principle, *n* = 104); 920 dribbling (1st principle, *n* = 256; 2nd A principle = 371, 2nd B principle, *n* = 215; 3rd principle, *n* = 78); and 342 shooting (3rd principle, *n* = 342) actions occurred. For 2vs2 SSCG, a total of 1087 passing (1st principle, *n* = 418; 2nd A principle = 155, 2nd B principle, *n* = 396; 3rd principle, *n* = 55); 1044 dribbling (1st principle, *n* = 318; 2nd A principle = 235, 2nd B principle, *n* = 277; 3rd principle, *n* = 214); and 421 shooting (3rd principle, *n* = 421) actions occurred.

**Figure 3:**
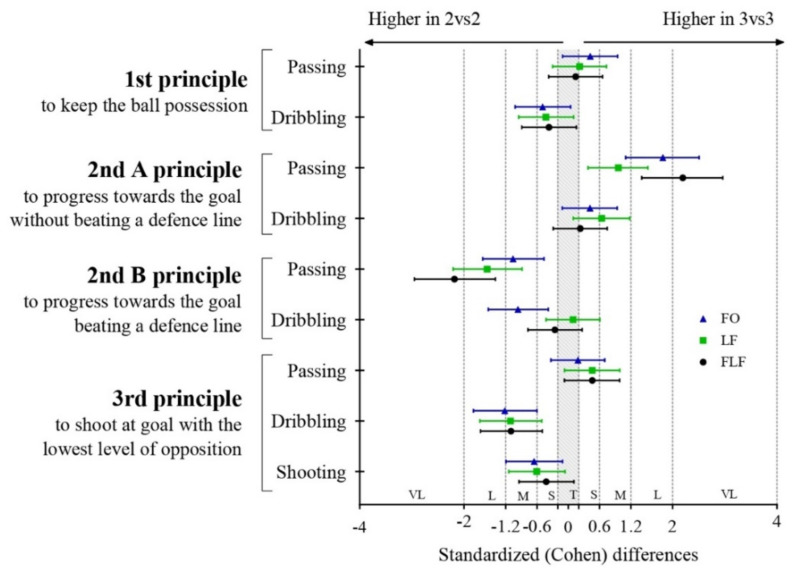
Standardized Cohen differences for the pairwise comparations between 2vs2 and 3vs3 for each action and game principle considered.

The descriptive and inferential analysis between actions per minute per player developed in two small-sided and conditioned games (2vs2–3vs3) according to the floater positioning (task constraint) and the game principle (GP) presented in Table 1. Additionally, Figure 3 shows the standardized (Cohen) differences for the pairwise comparations.

Non-significant differences were identified for passing and dribbling actions in the 1st principle (BP) for any task constraints between both SSCG. According to passing actions in 2nd A principle (P), the results showed significantly higher values in 3vs3 than in 2vs2 SSCG in FO (mean differences [95% confidence interval]; 3.1 [2.2, 4.1], *p* < 0.01, large ES), LF (2.4 [1.1, 3.7], *p* < 0.01, moderate ES) and FLF (4.9 [3.8, 6.1], *p* < 0.01, large ES). Regarding dribbling actions in 2nd A principle (P), results showed significantly higher values in 3vs3 than in 2vs2 SSCG in LF (1.3 [0.2, 2.4], *p* < 0.05, moderate ES).

When considering the passing actions in 2nd B principle (PDL), results showed significantly higher values in 2vs2 than in 3vs3 SSCG in FO (−1.5 [−2.2, −0.7], *p* < 0.01, moderate ES), LF (−3.3 [−4.4, −2.2], *p* < 0.01, large ES), and FLF (−3.5 [−4.4, −2.7], *p* < 0.01, very large ES). Regarding dribbling actions in 2nd B principle (PDL), results showed significantly higher values in 2vs2 than in 3vs3 SSCG in FO SSCG (−1.6 [−2.5, −0.7], *p* < 0.01, moderate ES).

For passing actions in the 3rd principle (S), no significant difference was identified. For dribbling actions performed in 3rd principle (S), results showed significantly higher values in 2vs2 than in 3vs3 SSCG in FO (−1.4 [−2.0, −0.8], *p* < 0.01, large ES), LF (−1.4 [−2.1, −0.7], *p* < 0.01, moderate ES) and FLF (−0.8 [−1.2, −0.4], *p* < 0.01, moderate ES). Finally, for the shooting actions in 3rd principle (S), results showed significantly higher values in 2vs2 than in 3vs3 SSCG in FO (−1.2 [−2.2, −0.2], *p* < 0.05, moderate ES) and LF (−1.1 [−2.1, −0.1], *p* < 0.05, moderate ES).

## 4. Discussion

This study aimed to analyse the manipulation of the number of players (Gk + 3vs3 + Gk and Gk + 2vs2 + Gk) and floater positioning on youth players’ technical–tactical offensive actions according to game principles. The highest values of passing were observed in the 3vs3 SSCG, where most dribbles and shots occurred in the 2vs2 SSCG. These results seem to indicate that the number of players per team as a task constraint can influence players and teams’ possibilities for action, and consequently their tactical behaviour. One of the first constraints that coaches need to consider when designing the practice tasks is the number of players involved [34]. When the goal is to create passing lines and maintain ball possession through passing, the 3vs3 SSCG should be used whereas if the focus is on dribbling and shooting, the 2vs2 situation can ensure a greater number of these actions. Furthermore, the manipulation of the number of players constrains not only the actions per se but the emergence of each action in relation to the game principles that support different purposes of the teams [35]. However, further studies are required with more participants from different levels of practice to generalize our results.

### 4.1. First Game Principle (1st = to Keep Possession)

With regard to the first game principle (BP), no significant difference was observed between the 2vs2 and the 3vs3 SSCG or the addition of floaters in the side or final line of the field. Contrary to previous research [15], a different number of players or floaters seems not to influence the number of passing or dribbling actions by players to maintain ball possession. Thus, a link between the goal and the manipulations promoted should be considered to understand the impacts of such manipulations on players’ and teams’ tactical behaviour [36].

### 4.2. Second Game Principle (2nd = to Progress towards the Goal)

Regarding the second game principle, two different categories were considered: 2nd A principle—to progress towards the goal without beating a defensive line (P) and 2nd B principle—to progress towards the goal by beating a defensive line (PDL).

Results of the 2nd A principle (P) revealed significantly higher values of passing in for the 3vs3 compared to the 2vs2 situation when players try to progress towards the goal without beating a defensive line in all experimental conditions (FO, FLF and LF). Thus, the number of players per team might be more determining for the emergence of progression without breaking a defensive line compared to the presence or absence of floaters. In agreement with previous research, the use of 3vs3 could be considered a more balanced defensive structure of play, defined by two defensive lines, not allowing an easy effective progression. As Gonçalves et al. (2016) and Vilar et al. (2014) pointed out, manipulating the number of players per team stimulates the emergence of new play patterns that support the emergence of different individual action possibilities for both attacking and defending players. Thus, it could be that in the 2vs2 the number of passing possibilities of the attacking team is limited (specifically, only one), so the defending team increases the pressure on the attacking players and the possibilities to do successful penetration (i.e., beating a defensive line) increases too. On the contrary, in the 3vs3 the defending team could retreat its position on the field by decreasing the distance between teammates and their own goal. As Pizarro et al. (2021) pointed out, when the defending team retreats its position, the distance between attacking and defending players increases and consequently, the probability of developing passing actions without beating the line increases. Furthermore, in 2vs2 teams, there is only one defensive line, which affords more advantage to progress, compared to the two existing defensive lines of the 3vs3 conditions that allows a better space equilibrium. Indeed, when a team has more players, the game is more positional and less variable, increasing the balance between teams [36].

No significant differences were observed for dribbling except for the condition LF, which revealed a higher number of dribbling actions favouring 3vs3. For the other side, the floater in the side-line allows more opportunities for dribbling in 2vs2. In line with previous research, the addition of the floater probably promoted a retreat of defenders on the field to guarantee the protection of space near the goal. Usually, when playing against unfavourable numerical relationships, the defender tends to decrease the space for action [27], maintaining the space equilibrium between defensive lines, not allowing passing actions, but inviting more 1vs1 dribbling situations [20,37]. Due to the 3vs3 structure allowing more than one defensive line, usually, such dribbling actions also do not afford the possibility to beat defensive lines.

Conversely, regarding the 2nd B principle (PDL), results revealed significantly higher values of passing in favour of 2vs2 when players try to progress towards the goal beating a defensive line in all experimental conditions (FO, FLF and LF). Interestingly, the effect tends to increase with the addition of floaters. With the increase in floaters, the number of passing actions that beat defensive lines in the 2vs2 conditions tends to increase in comparison with the 3vs3 conditions. In line with previous assumptions, the use of fewer defensive players decreased the number of defensive lines, increasing the need for each player to mark the opponent to maintain the spatial-temporal relations not to allow progression. It opens new possibilities to increase the mobility of attacking players to create passing lines for progression [38]. The addition of floaters promoted a numerical unbalance between teams, giving an advantage to attacking teams and allowing them to progress on the field, and consequently putting less pressure from defenders on the ball carrier, opening up more passing lines to the floaters [38]. The use of floaters in the final line in particular increases the number of passing lines and defenders attracted to the ball and seems to promote higher spatial unbalance for the emergence of passing opportunities.

Regarding dribbling actions, higher values were obtained in favour of 2vs2 when players try to progress towards the goal, beating a defensive line without the presence of floaters. In line with previous research, the absence of floaters and the small number of players (2vs2) seems to promote the emergence of 1vs1 situations, thus enabling the attacking players to perform more dribbling actions towards the opposite goal and beating a defensive line [20,37]. As previously stated, the addition of floaters tends to decrease the pressure of defenders on a ball carrier’s possibilities for passing actions instead of possibilities for dribbling [39].

### 4.3. Third Game Principle (3rd = to Shoot at Goal with the Lowest Level of Opposition)

Concerning the third game principle, only the dribbling and shooting revealed significant differences between conditions. No significant differences were observed for passing actions. The emergence of passing actions that support the shoot is quite similar for both conditions used, revealing the lower values of actions to support shooting.

The analysis of dribbling actions revealed significant differences in all the experimental conditions. Specifically, significantly higher values were obtained in favour of 2vs2 in comparison with 3vs3. Despite defenders in both seeking to maintain their position between the ball and the goal, not allowing a misalignment between the ball and the goal [40], variability in the attacking players’ relations with opponents and the ball is attributed to their constant explorative performances as they seek to break the symmetry with the defending players because of creating opportunities for scoring goals [41]. However, the explorative behaviours of the attacking team take place under the constraints imposed by the defending team. As noted, the defensive team tries to maintain spatiotemporal relations with the offensive team. In contrast, the offensive team attempts to disrupt the status quo at opportune times by advancing their position on the field, reaching the free attacking player, and finding chances for goal-scoring possibilities [42]. Therefore, the relevant issue is how players change their exploratory behaviours that disrupt the status quo: in 3vs3 through passing actions and in 2vs2 through dribbling and shooting.

### 4.4. Study Limitations and Future Research

As this research only involved male futsal players under 19, the generalization of findings to more diverse samples is limited. Additionally, the small sample size may refrain from achieving more robust inferences. Future research should overcome these issues and utilize players of varying ages, ability, and gender. On the other hand, this intervention was carried out in a natural context, where some contextual variables are challenging to control.

## 5. Conclusions and Practical Implications

This study has shown that manipulating the number of players (Gk + 3vs3 + Gk and Gk + 2vs2 + Gk) and floater positioning influence players´ technical–tactical behaviours in 3vs3 and 2vs2 SSCG. In the 2vs2, players perform more dribbling and shooting actions than in the 3vs3, where players developed more passing actions. However, these results vary depending on the game principle analysed. Specifically, 3vs3 is associated with passing and dribbling action to progress towards the goal without beating a defensive line, while 2vs2 is associated with passing and dribbling actions aimed at beating a defensive line. It probably happens because the defending team in 3vs3 form a zonal defence, necessitating the prioritization of avoiding creating penetrative passing lines and shooting at goal and increasing the pressure on the attacking players. Thus, within 2vs2 there seems to be more opportunities for 1vs1. According to the development steps, the overall results stress that the 2vs2 seems to highlight individual actions even with the presence of floaters, while the 3vs3 highlights more relational actions and collective tactical behaviours. However, as results have shown, there are differences between the individual actions developed according to the SSCG and the game principle. According to the main objective of training sessions, such information may support coaches in designing training tasks by manipulating task constraints (number of players and floaters that should be assigned to each goal).

## Figures and Tables

**Table 1:** Descriptive (Mean ± SD) and inferential analysis of the considered variables according to the SSCG formats.

Game Principle	Actions	Constraints	SSCG		Mean Difference with 95% CI	Effect Size
			2vs2	3vs3		
1st	Passing	Floaters Off	4.0 ± 2.5	5.1 ± 2.7	1.1 [−0.3, 2.5]	Unclear
		Lateral Floaters	4.3 ± 3.4	4.9 ± 2.2	0.6 [−0.9, 2.1]	Unclear
		Final Lines Floaters	3.0 ± 1.6	3.2 ± 1.7	0.2 [−0.6, 1.1]	Unclear
	Dribbling	Floaters Off	3.6 ± 2.0	2.6 ± 2	−1.0 [−2.0, 0.1]	Small
		Lateral Floaters	2.6 ± 1.7	2.0 ± 1.5	−0.7 [−1.6, 0.2]	Small
		Final Lines Floaters	3.1 ± 2.0	2.2 ± 2.3	−0.8 [−1.9, 0.3]	Unclear
2nd A	Passing	Floaters Off	0.8 ± 0.8	4.0 ± 2.3	3.1 [2.2, 4.1] *	Large
		Lateral Floaters	2.2 ± 2.0	4.7 ± 2.9	2.4 [1.1, 3.7] *	Moderate
		Final Lines Floaters	0.8 ± 0.9	5.9 ± 2.9	4.9 [3.8, 6.1] *	Very Large
	Dribbling	Floaters Off	2.9 ± 1.6	3.9 ± 2.9	1.0 [−0.3, 2.2]	Unclear
		Lateral Floaters	1.7 ± 1.4	3.1 ± 2.5	1.3 [0.2, 2.4] *	Moderate
		Final Lines Floaters	2.1 ± 1.3	2.5 ± 2.3	0.4 [−0.5, 1.4]	Unclear
2nd B	Passing	Floaters Off	2.7 ± 1.6	1.1 ± 1.1	−1.5 [−2.2, −0.7] *	Moderate
		Lateral Floaters	4.3 ± 2.8	1.1 ± 1.0	−3.3 [−4.4, −2.2] *	Large
		Final Lines Floaters	4.7 ± 2.0	1.2 ± 1.2	−3.5 [−4.4, −2.7] *	Very Large
	Dribbling	Floaters Off	3.5 ± 1.8	1.9 ± 1.5	−1.6 [−2.5, −0.7] *	Moderate
		Lateral Floaters	1.8 ± 1.5	1.9 ± 1.7	0.1 [−0.7, 1.0]	Unclear
		Final Lines Floaters	2.2 ± 2.1	1.8 ± 1.3	−0.4 [−1.4, 0.5]	Unclear
3rd	Passing	Floaters Off	0.7 ± 0.9	0.9 ± 1.1	0.2 [−0.3, 0.7]	Unclear
		Lateral Floaters	0.6 ± 0.8	1.0 ± 0.9	0.4 [−0.1, 0.8]	Small
		Final Lines Floaters	0.3 ± 0.5	0.6 ± 0.9	0.4 [−0.1, 0.8]	Small
	Dribbling	Floaters Off	2.1 ± 1.4	0.6 ± 0.8	−1.4 [−2.0, −0.8] *	Large
		Lateral Floaters	2.2 ± 1.5	0.8 ± 1.0	−1.4 [−2.1, −0.7] *	Moderate
		Final Lines Floaters	1.3 ± 0.9	0.4 ± 0.6	−0.8 [−1.2, −0.4] *	Moderate
	Shooting	Floaters Off	4.0 ± 1.9	2.7 ± 1.8	−1.2 [−2.2, −0.2] *	Moderate
		Lateral Floaters	3.8 ± 1.5	2.8 ± 1.9	−1.1 [−2.1, −0.1] *	Moderate
		Final Lines Floaters	3.7 ± 1.6	2.9 ± 1.7	−0.7 [−1.6, 0.2]	Small

* *p* < 0.05. Abbreviations: 1st = to keep possession; 2nd A = to progress towards the goal without beating a defensive line; 2nd B = to progress towards the goal beating a defensive line; 3rd = to shoot at goal with the lowest level of opposition.
